# Embolization of visceral arterial aneurysms: Simulation with 3D-printed models

**DOI:** 10.1177/1708538119900834

**Published:** 2020-01-19

**Authors:** Eisuke Shibata, Hidemasa Takao, Shiori Amemiya, Kuni Ohtomo, Osamu Abe

**Affiliations:** 1 Department of Radiology, The University of Tokyo, Graduate School of Medicine, Tokyo, Japan; 2 International University of Health and Welfare, Tochigi, Japan

**Keywords:** Simulation, 3D-printed model, endovascular treatment, aneurysm

## Abstract

**Objectives:**

The present technical article aimed to describe the efficacy of three-dimensional (3D)-printed hollow vascular models as a tool in the preoperative simulation of endovascular embolization of visceral artery aneurysms.

**Methods:**

From November 2015 to November 2016, four consecutive endovascular treatments of true visceral artery aneurysms were preoperatively simulated with 3D-printed hollow models. The mean age of the patients (one male and three females) was 54 (range: 40–71) years. Three patients presented with splenic artery aneurysm and one with anterior pancreaticoduodenal artery aneurysm. The average diameter of the aneurysms was 16.5 (range: 10–25) mm. The 3D-printed hollow models of the visceral artery aneurysms and involved arteries were created using computed tomography angiography data of the patients. After establishing treatment plans by simulations with the 3D-printed models, all patients received endovascular treatment.

**Results:**

All four hollow aneurysm models were successfully fabricated and used in the preoperative simulation of endovascular treatment. In the preoperative simulations with 3D-printed hollow models, splenic aneurysms were embolized with coils and/or *n*-butyl-2-cyanoacrylate to establish the actual treatment plans, and a small arterial branch originating from an anterior pancreaticoduodenal artery aneurysm was selected to obtain feedback regarding the behavior of catheters and guidewires. After establishing treatment plans by simulations, the visceral artery aneurysms of all patients were successfully embolized without major complications and recanalization.

**Conclusions:**

Simulation with 3D-printed hollow models can help establish an optimal treatment plan and may improve the safety and efficacy of endovascular treatment for visceral artery aneurysms.

## Introduction

Recently, interventional radiology has been considered the primary option for the treatment of visceral arterial aneurysms (VAAs) that have high risks of rupture. The treatment plan is generally established using anatomical information obtained from computed tomography (CT) angiography. The safety and efficacy of endovascular treatment depend mainly on the vascular anatomy and operator’s experience. However, knowledge of the anatomy alone is not sufficient to accurately predict the behavior of catheters, guidewires, and embolic agents during endovascular procedures, and the selection of optimal endovascular devices is also important for a successful treatment.

Three-dimensional (3D) printing, also known as rapid prototyping or additive manufacturing, is a rapidly developing technology that has been applied to human anatomical models. Among the various methods used for this technology, fused deposition modeling (FDM) is one of the most rapid and least expensive methods.^1^ Previous reports have shown the accuracy and precision of fabricated 3D-printed hollow models created using CT angiography data.^2–4^ However, few studies have applied 3D-printed hollow models in the preoperative simulation of endovascular treatment for VAAs.^5^

This report described the preoperative simulation of endovascular treatment for VAAs in four consecutive patients using 3D-printed hollow models.

## Materials and methods

### Patients

From November 2015 to November 2016, four consecutive patients with true VAAs received endovascular treatments at our department after performing simulations with 3D-printed hollow models. The mean age of the patients (one male and three females) was 54 (range: 40–71) years. Three patients presented with splenic artery aneurysm and one with anterior pancreaticoduodenal artery aneurysm. The average diameter of the aneurysms was 16.5 (range: 10–25) mm. None of the patients presented with symptoms of aneurysms (Table 1). This study was approved by the ethical committee of our institution.

**Table 1.** Characteristics of the patients and aneurysms and treatment outcomes.

Patient	Age (years)	Sex	Site of aneurysm	Aneurysm diameter (mm)	Embolic material	Technical Success	Follow-up (months)	Recanalization
1	71	F	Splenic artery	25	NBCA and metallic coils	Yes	45	No
2	55	F	Anterior pancreaticoduodenal artery	10	metallic coils	Yes	44	No
3	52	M	Splenic artery	15	NBCA and metallic coils	Yes	37	No
4	40	F	Splenic artery	15	NBCA and metallic coils	Yes	36	No

NBCA: *n*-butyl-2-cyanoacrylate.

### Model fabrication^2–4,6–8^

CT angiography was performed using either a 320-detector CT scanner (Aquilion ONE; Toshiba, Tochigi, Japan) or an 80-detector CT scanner (Aquilion PRIME; Toshiba). The scanning parameters were as follows: tube voltage, 120 kV; tube current, 141–169 mA (depends on bodily habitus); pixel size, 0.586 × 0.586 to 0.683 × 0.683 mm; slice interval, 0.5–0.8 mm; and slice thickness, 0.5–1 mm. Iopamidol (Iopamiron 370; Bayer, Osaka, Japan) or iohexol (Omnipaque 350; Daiichi Sankyo, Tokyo, Japan) was injected at a rate of 3.2 mL/s. The volume of contrast medium was based on the patient’s weight. CT angiography scanning was started manually by monitoring the CT values of the aorta with bolus tracking. The acquired data were exported to an image analysis workstation (AZE Virtual Place; AZE Ltd, Tokyo, Japan). The mask images of the aneurysms and involved arteries were created using 3D object selection and region cutting with shaded surface display (SSD). The distribution of CT values (histogram) for each aneurysm was calculated, and the threshold for SSD was calculated as follows while considering the partial volume effect: (peak distributed CT intensity + 80)/2. We assumed a surrounding tissue CT intensity of 80 HU. The calculated thresholds were 208 HU in patient 1, 243 HU in patient 2, 149 HU in patient 3, and 193 HU in patient 4. The mask images were then binarized (0 or 255) and inverted to obtain hollow vascular models. The inverted mask images were converted to stereolithography (STL) files using an open source medical imaging software (Osirix) and 3D surface rendering with a threshold of 128. Hollow aneurysm models were fabricated with nylon material and a layer thickness of 200 µm using an FDM-type 3D printer (CubePro; 3D systems, Rock Hill, SC, USA).

### Simulation

All simulations were performed under fluoroscopic guidance in the angiography room. Each model was connected to a tube and sheath and was filled with saline. All angiography procedures were performed with contrast media under a manually created flow or under balloon occlusion.

## Results

**Figure 1.**
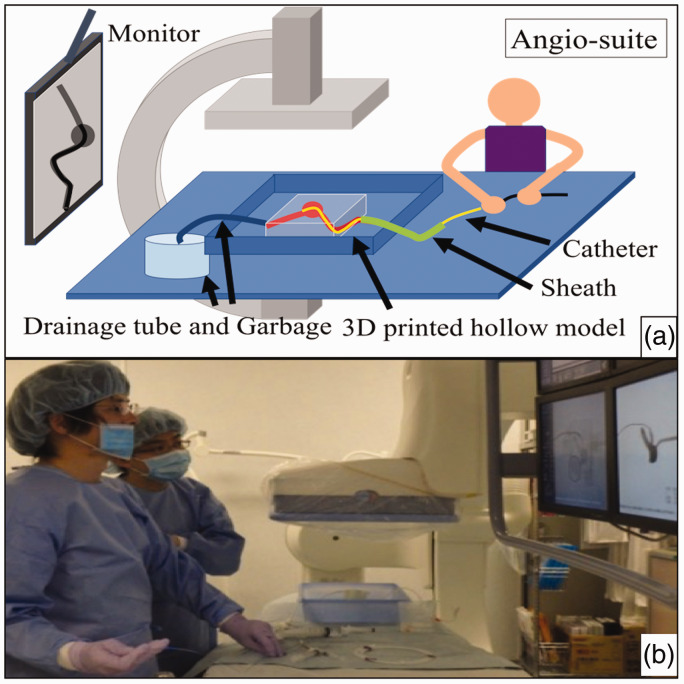
Simulation with three-dimensional (3D) printed hollow models. (a) Schematic diagram showing that the sheath was connected to a 3D-printed hollow model and the catheters were inserted via the sheath to the aneurysm. (b) Simulation performed in the angiography room. The operator can obtain feedback about the treatment plan before the actual treatment.

Each 3D-printed hollow model was successfully fabricated and used in the preoperative simulation of endovascular treatment (Figure 1). All patients were successfully treated without major complications and recanalization.

### Patient 1

**Figure 2.**
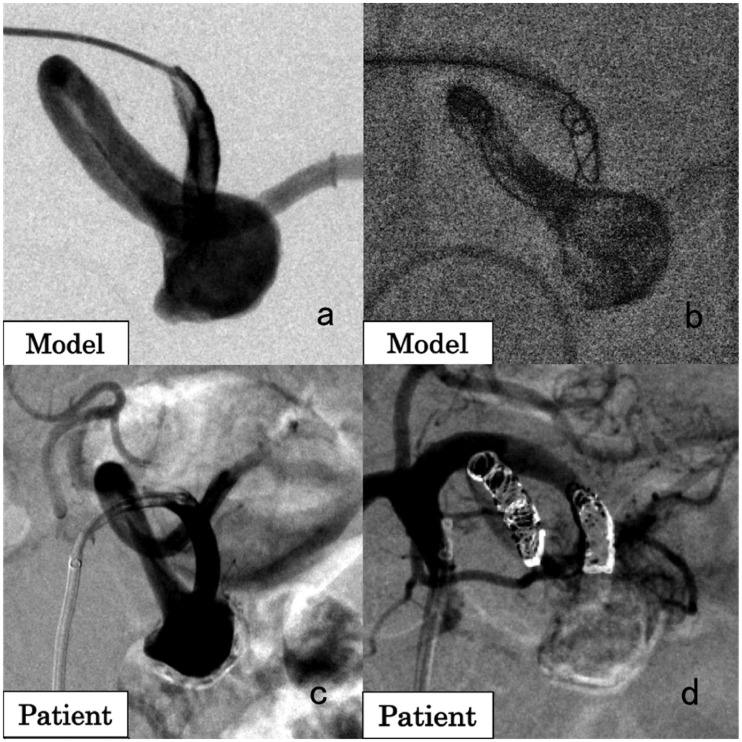
A 71-year-old female patient with a splenic artery aneurysm (SAA). (a) Angiographic image of the 3D-printed model. (b) Simulation of the SAA embolization. Under balloon occlusion, the artery distal to the SAA was embolized with coils, followed by injection of *n*-butyl-2-cyanoacrylate (NBCA) and 5% glucose to make NBCA foamy. Finally, the artery proximal to the SAA was embolized with coils. (c) Angiographic image in the patient showing the SAA. (d) Angiographic image of the splenic artery after embolization. No blood flow was observed in the SAA.

A 71-year-old female patient had a 25 mm splenic artery aneurysm (SAA) located in the distal segment of the main splenic artery. A 3D-printed model was created for preoperative simulation (Figure 2(a)). Coils were placed in the vessel distal to the SAA under balloon occlusion. *n*-butyl-2-cyanoacrylate (NBCA) (NBCA: Lipiodol = 1:1) was injected into the SAA, followed by 5% glucose to make NBCA foamy and to fill the entire SAA with a small amount of NBCA. Coils were then placed in the vessel proximal to the SAA (Figure 2(b)). In the actual treatment of the patient (Figure 2(c)), with a 5.2 Fr balloon catheter and a 2.5 Fr microcatheter, interlocking detachable coils (Interlock Fibered IDC 2 D Helical, 5 mm/15 cm × 3, 4 mm/15 cm × 1; Boston Scientific, MA, USA) were placed in the vessel distal to the SAA under flow control. For aneurysmal packing, the same amount of NBCA was used, followed by injection with 5% glucose. In the vessel proximal to the SAA, coils (Interlock Fibered IDC 2 D Helical, 4 mm/15 cm × 1; Tornado, 6/2 mm × 5, 4/2 mm × 3; Cook Medical, IN, USA) were placed (Figure 2(d)). No major complication was noted, and there was no recanalization 45 months after treatment.

### Patient 2

**Figure 3.**
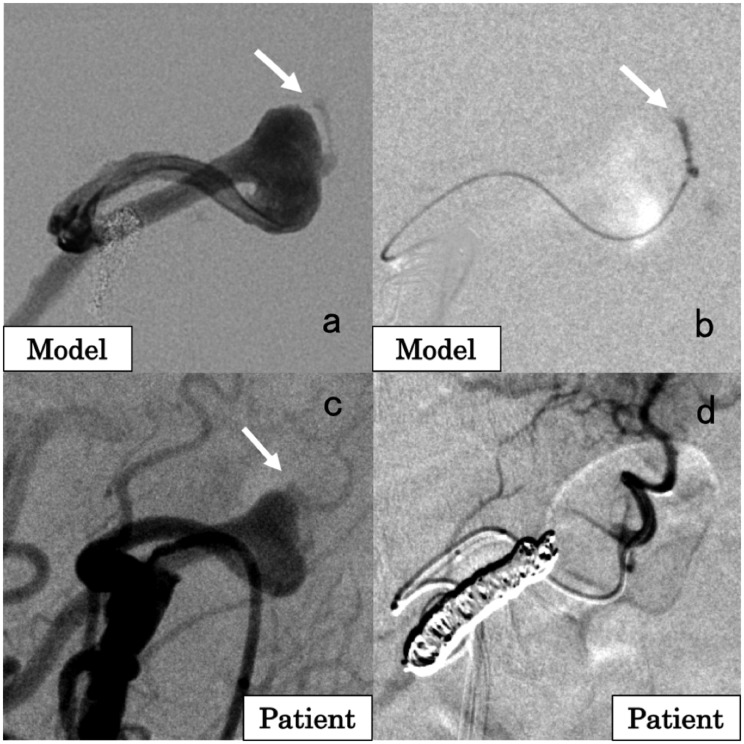
A 55-year-old female patient with an anterior pancreaticoduodenal artery (APDA) aneurysm. (a) Angiographic image of the 3D-printed model. The arrow indicates a small arterial branch originating from the aneurysm. (b) Selection of the small branch originating from the APDA aneurysm in the simulation. This procedure was the main purpose of the simulation because the selection of a small artery from the aneurysm is sometimes difficult. (c) Angiographic image of the superior mesenteric artery (SMA) in the patient showing that the aneurysm was located in the APDA. The arrow indicates the small branch originating from the aneurysm. (d) Angiographic image of the small branch originating from the APDA aneurysm after coil embolization of the artery distal to the aneurysm.

A 55-year-old female patient had a 10 mm anterior pancreaticoduodenal artery (APDA) aneurysm with one small branch that fed the pancreas. CT scan revealed severe stenosis of the celiac artery caused by the median arcuate ligament. Thus, the treatment plan was to embolize the APDA aneurysm via the superior mesenteric artery. A 3D-printed model was fabricated for simulation. In this case, the focus of simulation was to select the small artery originating from the aneurysm because the selection of this artery was considered difficult. In the simulation (Figure 3(a)), a 2.5 Fr microcatheter and a 4 Fr catheter were advanced to the APDA using a sheathless guiding catheter. The selection of the small vessel originating from the aneurysm was achieved by shaping the microguidewire (Figure 3(b)). In the actual treatment of the patient (Figure 3(c)), after the embolization of the vessel distal to the aneurysm with coils (Interlock Fibered IDC 2 D Helical, 6 mm/20 cm × 1; Tornado, 6/2 mm × 5, 5/2 mm × 2, 4/2 mm × 1), the small branch originating from the aneurysm was successfully selected (Figure 3(d)), and coils were placed (Tornado, 4/2 mm × 3, 3/2 mm × 1). The vessel proximal to the aneurysm was then embolized with coils (Interlock Fibered IDC 2 D Helical, 6 mm/20 cm × 3; Tornado, 6/2 mm × 5, 4/2 mm × 3).^9^ No major complication was observed, and there was no recanalization 44 months after treatment.

### Patient 3

**Figure 4.**
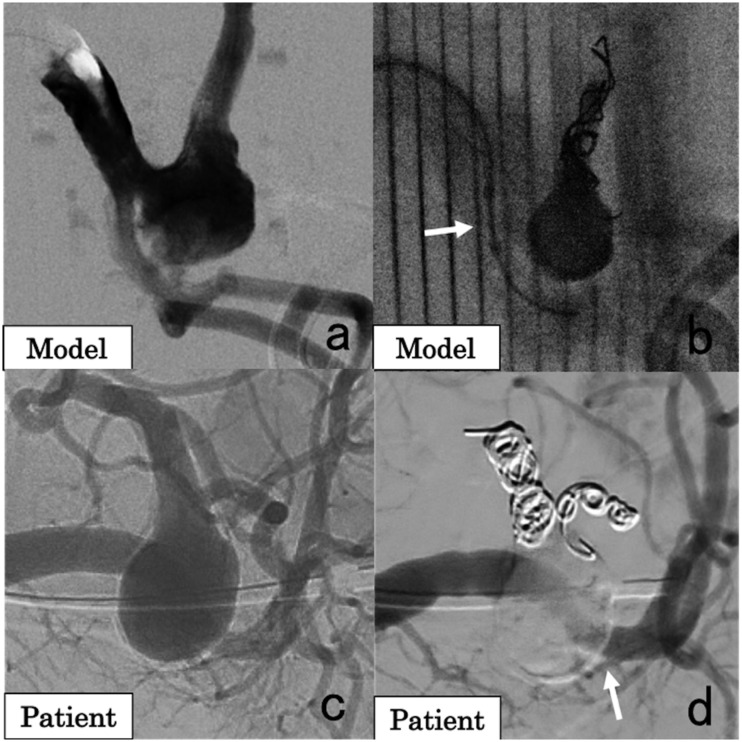
A 52-year-old male patient with a splenic artery aneurysm (SAA). (a) Angiographic image of the 3D-printed model showing that the SAA was located in the proximal part of the upper branch of the splenic artery. The SAA has a short proximal neck. (b) Simulation of coil embolization distal to the aneurysm, followed by injection of *n*-butyl-2-cyanoacrylate (NBCA) under balloon occlusion (arrow) at the proximal neck of the aneurysm. (c) Angiographic image of the splenic artery in the patient showing the SAA in the proximal part of the upper branch of the splenic artery. (d) Angiographic image after embolization of the SAA with coils and NBCA. The inferior branch of the splenic artery was preserved (arrow).

A 52-year-old male patient had a 15 mm SAA with a short proximal neck located at the proximal part of the upper branch of the splenic artery. A 3D-printed model was created for preoperative simulation. In the simulation (Figure 4(a)), the vessel distal to the aneurysm was first embolized with coils. Due to insufficient space for isolation with coils in the proximal neck of the aneurysm, NBCA was injected into the aneurysm and the short proximal neck under flow control with a balloon catheter, which was advanced to the inferior branch of the splenic artery and was adjusted to cover the proximal neck of the aneurysm in the upper branch of the splenic artery (Figure 4(b)). In the actual treatment (Figure 4(c)), the vessels distal to the aneurysm were embolized with coils (Interlock Fibered IDC 2 D Helical, 7 mm/10 cm × 1, 6 mm/20 cm × 1; Interlock Fibered IDC VortX Diamond, 4 mm/2 mm × 1; Tornado, 5/2 mm × 2, 4/2 mm × 1, and 3/2 mm × 1). A 5.2 Fr balloon catheter was advanced to the inferior branch of the splenic artery and was adjusted to cover the neck of the aneurysm in the upper branch of the splenic artery by balloon expansion. A tri-axial system comprising a 4 Fr catheter and 2.7 Fr and 1.9 Fr microcatheters was inserted from the other femoral artery and used to fill the aneurysm with NBCA (NBCA:Lipiodol = 1:1) under balloon occlusion (Figure 4(d)). No major complication was observed, and there was no recanalization 37 months after treatment.

### Patient 4

**Figure 5.**
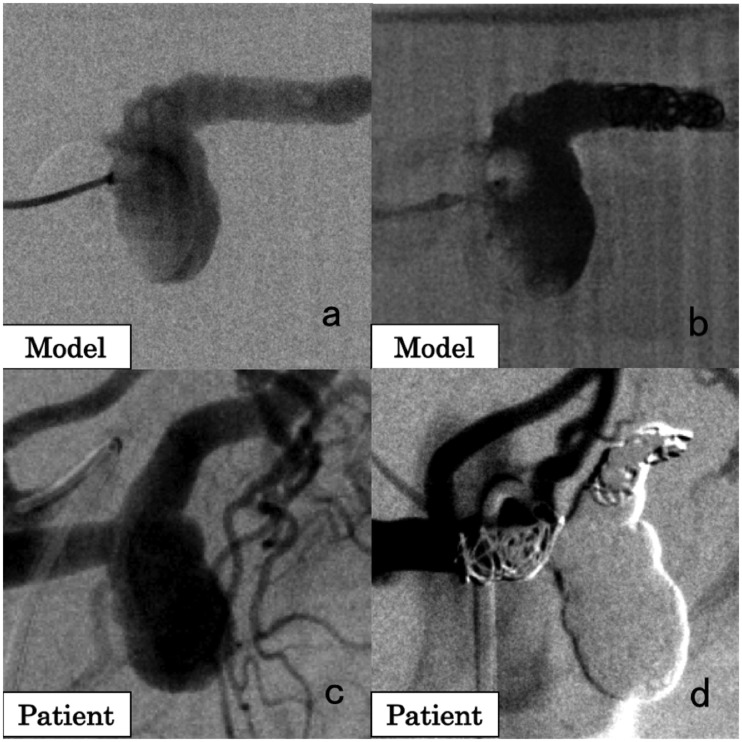
A 40-year-old female patient with a splenic artery aneurysm (SAA). (a) Angiographic image of the 3D-printed model showing the SAA. (b) Angiographic image in the simulation showing injection of *n*-butyl-2-cyanoacrylate (NBCA) after distal coil embolization of the SAA. (c) Angiographic image of the splenic artery in the patient showing the SAA. (d) Angiographic image of the splenic artery after embolization of the SAA with NBCA and coils.

A 40-year-old female patient had a 15 mm SAA. The aneurysm was located proximal to the main splenic artery and had no small branches. A 3D-printed model was created for preoperative simulation (Figure 5(a)), and the SAA was filled with NBCA after distal coil embolization (Figure 5(b)). In the actual treatment (Figure 5(c)), distal embolization was performed with coils (Interlock Fibered IDC 2 D Helical, 7 mm/10 cm × 1, 5 mm/8 cm × 1; Tornado, 5/2 mm × 1, 4/2 mm × 1, 3/2 mm × 6). NBCA (NBCA: Lipiodol = 1:1) was injected to the aneurysm. Coils (Interlock Fibered IDC 2 D Helical, 8 mm/20 cm × 1; Tornado, 6/2 mm × 6) were then placed in the vessel proximal to the aneurysm (Figure 5(d)). No major complications were observed, and there was no recanalization 36 months after embolization.

## Discussion

Three-dimensional printing is a useful tool in clinical practice and medical education because the 3D models can facilitate clear visualization of the spatial relationships between anatomical structures. In terms of education, previous reports have shown that 3D printed models help residents, medical staff, and patients to better understand anatomical structures and diseases.^10–12^ In clinical practice, models have been used in the preoperative simulation of intracranial endovascular aneurysm coiling, cerebral aneurysm clipping, stent placement in the aorta, and CT-guided procedures.^13–16^ Weinstock et al. have reported that preoperative simulation with 3D-printed models can shorten operation time by optimizing procedures in children with intracranial arteriovenous malformations.^17^ However, only few reports have shown the use of 3D-printed hollow models in the preoperative simulation of endovascular treatments for VAAs.^5^ In our report, we used real catheters, guidewires, and embolic agents to embolize the 3D-printed hollow models of aneurysms, which were created using CT angiography data. Simulation that is highly similar to actual treatment of patients can allow physicians to gain accurate feedback about the procedure and to develop a safer and more successful treatment plan.

Individual variations of arteries are often detected in the treatment of abdominal aneurysms. In addition, tortuosity of arteries, such as the celiac artery, the superior mesenteric artery, and even the aorta can make endovascular treatment difficult. However, CT angiography cannot predict the real behavior of catheter guidewires during the procedure even if the operator has a detailed knowledge of the individual vascular structures based on 3D reconstructed data or 3D-printed models. Thus, feedback from simulation may improve safety and shorten the operation time. The preoperative simulation of endovascular treatment using 3D-printed hollow models is also useful in educating and training young interventionalists. The safety and efficacy of endovascular treatment depend mainly on vascular anatomy and operator’s experience. By simulation with 3D-printed models, real catheters, and guidewires, young physicians can develop their skills in the endovascular treatment of vascular diseases, thereby leading to shortened operation time, less radiation exposure to patients, improved safety, and high success rates.

Some tools that can be used for endovascular education and training are available. Clear tube models of the vessels can help in understanding the manipulation of endovascular devices by direct viewing. With the use of clear models, physicians and medical staff can learn about endovascular treatment without fluoroscopy and contrast media. In our report, simulations with 3D-printed models were performed under fluoroscopy in the angiography room to make the experience similar to treatments in actual clinical settings. Virtual reality (VR) simulation is also one of the new topics in medical education in the fields of endovascular treatment, such as endovascular aneurysm repair and lower limb endovascular interventions.^18,19^ The VR simulator can provide a realistic environment and automatically record all parameters, including procedure time, amount of contrast media used, and even contact of wires and catheters with vessel walls. However, the cost of simulation and maintenance is a problem. Our simulation with the 3D-printed models is not large scaled. It is less expensive and easier to apply than the VR simulation system.

There were limitations to our simulation. First, the 3D-printed hollow models were made of nylon, which has a different texture and elasticity from real arteries. Changes in the tortuous artery shapes during advancement of guidewires and catheters were not observed during simulation. In the actual treatment, the final decisions about the volume of NBCA or the number of coils were made based on real angiography findings. Second, the internal lumen of the models was not as smooth as that of real arteries. In previous studies, 3D-printed hollow models were created accurately and precisely with the FDM method.^2–4^ However, in this method, melted filaments are placed layer-by-layer, which may cause unevenness. Third, blood flow was not simulated in this study, because the focus was to obtain feedback about the performance and usability of catheters, guidewires and embolic agents, and because embolization with NBCA was performed under flow control by balloon occlusion.

We believe that simulation with 3D-printed models does not only help improve the skills or knowledge of medical staff about endovascular treatment without any risk to patients, but also decrease complication or failure rate with endovascular treatment. We hope that physicians can have easy access to simulation with 3D-printed models or virtual simulators in the future.

In conclusion, we demonstrated preoperative simulations of endovascular treatment for VAAs to establish treatment plans and improve the safety and success rate of endovascular treatment. Additional studies, particularly with statistical analyses, must be conducted to validate the effects of preoperative simulation with 3D-printed models on treatment outcomes of VAA embolization.

## Supplemental Material

VAS900834 Supplemental material - Supplemental material for Embolization of visceral arterial aneurysms: Simulation with 3D-printed models Click here for additional data file. Supplemental material, VAS900834 Supplemental material for Embolization of visceral arterial aneurysms: Simulation with 3D-printed models by Eisuke Shibata, Hidemasa Takao, Shiori Amemiya, Kuni Ohtomo and Osamu Abe in Vascular
