# Supplementary material for: Embolization of visceral arterial aneurysms: Simulation with 3D-printed models
Source: Vascular. 2020 Jan 19;28(3):259–66. doi: 10.1177/1708538119900834 (PMC7294531; doi:10.1177/1708538119900834)
Supplement: VAS900834 Supplemental material - Supplemental material for Embolization of visceral arterial aneurysms: Simulation with 3D-printed models [file VAS900834_Supplemental_material.pdf]

Embolization of visceral arterial aneurysms: simulation with 3D-printed models

Type of manuscript;

Technical article

Author;

Eisuke Shibata[1], Hidemasa Takao[1], Shiori Amemiya[1], Kuni Ohtomo[1,2],

Osamu Abe[1]

1) Department of Radiology, The University of Tokyo, Graduate School of Medicine,

   7-3-1 Hongo, Bunkyo-ku, Tokyo 113-8655, Japan

2) International University of Health and Welfare, 2600-1, Kitakanemaru, Ohtawara,

   Tochigi 324-8501, Japan

Acknowledgment of grant support;

This work was supported by a Grant-in-Aid for Scientific Research (A) 15H02553

from Japan Society for the Promotion of Science.

Kojimachi Business Center Building, 5-3-1 Kojimachi, Chiyoda-ku, Tokyo 102-0083

Telephone: +81-3-3263-1722

Full list of declarations;

All authors declared that they had no interest of conflict.

Corresponding author;

Eisuke Shibata

Department of Radiology, the University of Tokyo, Graduate School of Medicine, 7-3-1

Hongo, Bunkyo-ku, Tokyo 113-8655, Japan

Email: eisuke.shibata1130@gmail.com

Telephone: +81-3-3815-5411

Fax: +81-3-5800-8630
